# Decarbonizing energy: Evaluating fossil fuel displacement by renewables in OECD countries

**DOI:** 10.1007/s11356-024-33324-8

**Published:** 2024-04-17

**Authors:** Selin Karlilar Pata, Mehmet Balcilar

**Affiliations:** 1 Department of Economics, Hatay Mustafa Kemal University, Hatay, Türkiye; 2 Clinic of Economics, Azerbaijan State University of Economics (UNEC), Baku, Azerbaijan; 3 Department of Economics and Business Analytics, University of New Haven, 300 Boston Post Road, West Haven, CT 06516 USA

**Keywords:** Energy displacement, Renewable energy, Fossil fuels, Wind energy, Solar energy, Environmental sustainability

## Abstract

Energy transition to greener systems has been a focal point in climate policy agendas across countries as the negative environmental impacts of fossil fuel technologies have become more evident Displacing fossil fuels with clean energy alternatives in this regard is essential for meeting global climate objectives. In this context, the study analyzes the role of disaggregated renewable energy sources on fossil fuel displacement in 36 Organisation for Economic Cooperation and Development (OECD) countries in the period 2000–2020. The findings demonstrate a discernible trend in the displacement of fossil fuels by various forms of renewable energy sources. It is found that to effectively displace 1% of fossil fuels, it is necessary to achieve an average increase of 1.15% in renewable generation capacity. In addition, a one-to-one displacement of fossil fuels occurs with hydropower, demonstrating its higher level of competitiveness and effectiveness in displacing fossil fuels. Moreover, there is a partial displacement of fossil fuels by solar and wind power. These findings suggest that renewable energy sources are progressively advancing towards effectively displacing fossil fuels.

## Introduction

According to a report by the United Nations (United Nations (UN) 2022), the energy sector is a significant contributor to climate change, accounting for approximately 70% of global greenhouse gas (GhG) emissions. It is widely acknowledged that the use of fossil fuels for energy generation is one of the leading causes of GhG emissions and, consequently, climate change (Greiner et al. 2022). In addition to environmental considerations, the unequal distribution of fossil fuels raises concerns regarding energy security and efficiency, given their role in contemporary energy production systems (Martins et al. 2019). As a result, the continuous increase in GhG emissions due to global energy generation underscores the critical importance of implementing necessary actions for climate change mitigation. In this context, all UN Member States adopted the 2030 Agenda for Sustainable Development in 2015, which comprises 17 Sustainable Development Goals (SDGs). Notably, SDG-7 (Affordable and Clean Energy) and SDG-13 (Urgent Action to Combat Climate Change) have attracted significant attention in response to climate change caused by pollutant emissions. Furthermore, the 2021 COP26 United Nations Climate Change Conference reached a consensus on environmental objectives, which included achieving net-zero emissions by mid-century, mobilizing financing to promote clean energy adoption, and protecting natural resources (Arora and Mishra 2021). In this regard, replacing fossil fuels with clean energy alternatives, particularly renewables, is essential for realizing global climate objectives. Integrating renewable energy (RE) sources into the current energy system constitutes one of the most vital components of such decarbonization strategies (Soini et al. 2020).

Recently, substantial advancements in RE sources have been observed, indicating that the process of transitioning to renewables is well underway worldwide. On one hand, there has been a significant shift in the competitive equilibrium between renewables and fossil fuels from 2010 to 2021. The growth in RE generation capacities has resulted in a considerable reduction in costs associated with generating energy using RE technologies (IRENA 2018). In light of the current high prices of fossil fuels, the integration of RE in 2021 has saved global energy generation costs by approximately USD 55 billion in 2022. Furthermore, 62% of the total new renewable power generation capacity added globally in 2020 had lower energy costs than the cheapest source of fossil fuel-fired capacity. Since Q4 2021, fossil fuel costs have risen at a faster pace than RE costs, allowing renewables to sustain their competitive advantage (IRENA 2022a). Consequently, RE sources have become the primary low-cost option for power generation amid the present fossil fuel crisis.

On the other hand, renewables have achieved remarkable progress in the power sector, with capacity additions reaching new highs. Over the past decade, RE generation capacity increased by 130%, while that of fossil fuels increased by only 24%. Moreover, global renewable generation capacity has expanded nearly fourfold from 754 to 3064 GW between 2000 and 2021 (IRENA 2022b). In 2021, RE sources accounted for 81% of the total power generation capacity. Specifically, hydropower remains the largest RE source, comprising 40% of the overall installed capacity of renewables in 2021. Solar photovoltaic (PV) installations have experienced the most rapid growth among RE sources, demonstrating a 21-fold increase from 2010 to 2021. Wind power has also shown significant growth within the same time frame as solar power, with installations increasing more than fourfold. Additionally, other forms of RE sources, such as geothermal and bioenergy, have witnessed a considerable increase over the past decade, albeit on a small scale (IRENA 2022a, 2022b). In summary, RE sources are progressively displacing conventional energy sources on a global scale.

**Fig. 1:**
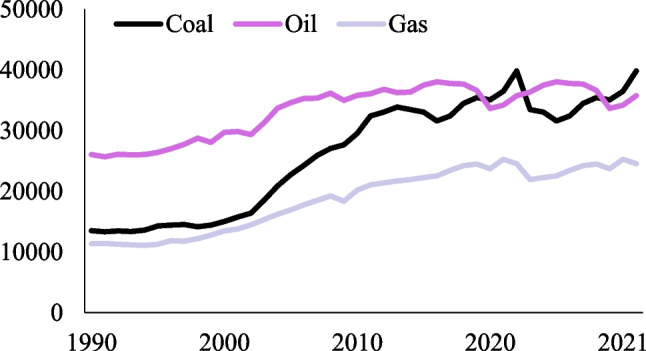
Non-renewable energy generation in OECD countries (Twh). Source: Our World in Data (2023)

Despite the notable progress achieved by renewable energy sources globally, fossil fuels continue to hold a prominent position in the energy sector of OECD countries. These nations contribute roughly 35% of global energy-related carbon emissions and produce twice the CO_2_ emissions per capita (8.9 t) compared to the global average (4.3 t) (IEA 2022a). Given these data, addressing environmental issues and transitioning to renewable energies have emerged as primary development strategies for OECD countries to maximize the advantages of clean technologies. Figure 1 shows the production of non-renewable energy in OECD countries.

Figure 1 shows that OECD countries have increased their non-renewable energy production over the period 1990–2021. Indeed, there has been an observable drop in their pace of increase during the past decade. Additionally, the production of gas in these nations has experienced a fall in recent years in comparison to coal and oil.

**Fig. 2:**
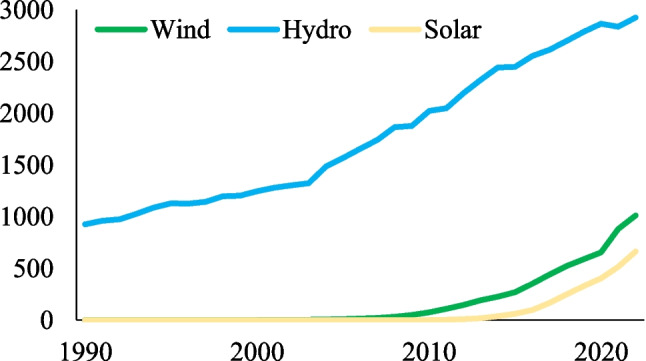
Renewable energy generation in OECD countries (Twh). Source: Our World in Data (2023)

Figure 2 illustrates the renewable energy generation in OECD countries. Hydropower is the RE source that OECD countries use extensively, as seen in Fig. 2. Moreover, the utilization of variable renewable energy sources like wind and solar power has significantly increased over the last decade. However, there is still a pathway for further development and improvement for these energy sources.

A majority of OECD countries have recently implemented environmental policies aimed at significantly increasing the share of RE within their energy mix. For instance, Japan has augmented its utilization of renewables and established a target of deriving 36–38% of its energy generation from renewable sources as part of its efforts to attain carbon neutrality by 2050. Moreover, as of 2016, Japan had become the second-largest solar energy producer among OECD countries (IEA 2020). In Australia, the proportion of renewables in total energy generation reached approximately 23.5% in 2021, as the country strives to sustain this progress until 2030. In Denmark, the annual share of variable energy sources, such as solar and wind power, exceeded 50% (REN21, 2022). Furthermore, RE generation in OECD Europe surpassed that of fossil fuel generation in both 2020 and 2019. This trend was particularly evident in countries such as France, Germany, Italy, and Spain, as they reaped the greatest benefits from the displacement of fossil fuels by renewables within OECD-Europe economies. These developments illustrate the rapid expansion of the RE sector across OECD-Europe, particularly in the domains of wind and solar power (IEA 2020; IRENA 2022a).

In summation, it is increasingly clear that the world is confronted with a significant threat posed by global warming. To alleviate its devastating consequences, a substantial reduction in the reliance on fossil fuels is imperative. In this context, low-carbon and renewable energy technologies have become a crucial component in global initiatives aimed at decreasing reliance on fossil fuels and saving CO_2_ emissions. Numerous countries have placed great emphasis on promoting the use of renewable energy sources as part of their environmental objectives, with the aim of achieving carbon neutrality. Given the imminent danger of global climate change, it is imperative that renewable energy sources not only increase their power generation but also replace fossil fuels to prevent a climatic catastrophe.

The first contribution of the present study is to address the following pivotal question: To what degree are RE sources capable of displacing fossil fuels, given the considerable challenges they present to the decarbonization process within the energy sector of OECD countries? Second, the study sample encompasses countries that accounted for the majority of global RE investment during the analyzed period. Third, while existing energy literature has examined the environmental impact of RE sources or fossil fuels, the topic of energy displacement remains relatively unexplored, warranting further investigation. This study represents a novel contribution to the field as it explores the possibility of disaggregated renewable energy sources displacing fossil fuels. It is crucial to determine the relative efficacy of different renewable technologies in replacing fossil fuels. Fourth, the current study employs the Common Correlated Effects Mean Group (CCE-MG) method on panel data from 36 OECD countries spanning from 2000 to 2020 to provide an empirical answer to the research question by assessing the displacement effect of renewables on fossil fuels. Lastly, having a clear understanding of which type of renewable energy is most effective in replacing fossil fuels is crucial for policymakers when it comes to refining environmental policies that can effectively combat climate change. Hence, this study offers robust evidence regarding the critical role renewables play in achieving climate change goals.

The structure of this paper is as follows: “Related literature” offers a summary of the relevant literature. “Data, model specification and methods” outlines the variables and the empirical model employed in this study, followed by a presentation of the empirical results in “Empirical results and discussion.” Finally, “Conclusion and policy implications” concludes the study and delineates potential policy implications.

## Related literature

The environmental consequences of excessive pollutant emissions resulting from fossil fuel use have led to the emergence of renewable energy as a viable and sustainable alternative, characterized by its low carbon footprint (Li et al. 2023). The transition towards clean energy sources promises considerable benefits by diminishing the adverse environmental impacts associated with fossil fuel-based energy production and consumption. In this context, a multitude of studies have presented empirical evidence that substantiates the emission-reducing impacts of RE sources as viable alternatives for fossil fuels (Deka et al. 2023; Kartal et al. 2023; Wang et al. 2023a), underscoring their significance in attaining decarbonization within the energy sector (Ulucak and Yucel 2021). Consequently, to effectively tackle the climate crisis, renewable energy sources must serve as a substitute for fossil fuels rather than merely facilitate a sustained rise in energy use.

The first strand of the literature concentrates on the interplay between RE sources and fossil fuels. For instance, Awerbuch and Sauter (2006) emphasize that RE investments displace fossil fuels, particularly natural gas and oil, in the United States (U.S.) and Europe, thereby mitigating economic losses resulting from the oil-GDP effect. Marques and Fuinhas (2011) explore the factors driving RE utilization in 24 selected European countries between 1990 and 2006; their research indicated that conventional energy sources hinder the deployment of RE. Narbel (2013), utilizing panel data on electricity generation in 107 countries, asserted that fossil fuel sources impede the diffusion of renewable technology. Pfeiffer and Mulder (2013) reveal that the abundance of fossil fuels and escalating energy consumption appear to negatively impact the quantity of non-hydro RE generated, concluding that non-hydro RE and fossil fuels are predominantly perceived as substitutes rather than complements to each other in numerous emerging economies. Assessing a sample of 38 countries, Aguirre and Ibikunle (2014) investigate the determinants of RE growth; their empirical findings indicated a negative correlation between RE growth and the contribution of coal, oil, and natural gas to electricity production. Prehoda and Pearce (2017) emphasize that replacing coal power plants with solar power in the U.S. yields significant environmental benefits and a clear path towards a more sustainable environment. Analogously, in the context of Portugal, Figueiredo et al. (2019) report that the coal power plant phase-out without additional power capacity installation results in a fivefold increase in energy imports, underscoring the critical role of renewable power capacity integration into the energy system. In this scenario, solar photovoltaics could potentially replace coal power plants. In contrast, Verdolini et al. (2018) identify a complementary relationship between fossil fuels and RE technologies in 26 OECD countries. Employing a large sample of countries, Greiner et al. (2022) discover that increasing RE production effectively curtails nuclear energy production; however, renewables tend to replace fossil fuels only modestly.

The second strand of literature emphasizes the technological advancements that hold promising potential for expanding RE capacities. Cutting-edge technology plays a crucial role in the integration of RE sources and enhances the overall capacity for electricity generation (Wang and Dong 2022; Yu et al. 2023). Popp et al. (2011) find that knowledge stocks exerted a small yet beneficial effect on RE capacity investments across 26 OECD countries. Verdolini and Galeotti (2011) demonstrate that augmented demand for energy-related technologies and increased knowledge stocks are positively associated with elevated levels of innovative activity. Hille and Oelker (2023) document that innovation activity, which has contributed to substantial cost efficiency improvements and is crucial for the sustained success of RE, would not have advanced as rapidly and fostered the growth of solar and wind capacities without policy support. They conclude that the influence of innovation on RE diffusion is predominantly policy-induced. Their study’s findings suggest that technological differences, particularly innovation, appear to exert a more significant impact on the development of solar capacities in comparison to wind capacities.

Conversely, within innovation research, patents are regarded as a reliable and statistically robust proxy for technological advancements and encompass an extensive body of technical knowledge (Johnstone et al. 2010; Zheng et al. 2021). RE sources are becoming more competitive in the market as a result of technological advancements that are advancing and disseminating energy-related patents (Wang et al. 2023b). In this context, Wurlod and Noailly (2018) underscore that green patenting activities are inversely related to energy intensity in OECD countries. Alexiou (2023) contends that implementing patent systems could potentially stimulate research and development efforts in the domain of RE, leading to considerable progress in advancing RE technologies. As a result, beneficial spillover effects may arise from the enhanced efficiency and cost-effectiveness of these technologies, subsequently augmenting their competitive edge relative to fossil fuel energy sources. Yang et al. (2022) observe that countries possessing greater green innovation capacity tend to exhibit higher efficacy in enacting renewable policies. Xin et al. (2023) conclude that technological innovation in RE promotes low-carbon development in China.

## Data, model specification and methods

The emphasis of this paper lies in examining the displacement effect of RE sources on fossil fuels. To achieve this objective, a panel dataset encompassing 36 OECD countries, with the exclusion of Iceland and Costa Rica due to data unavailability, is utilized for the period between 2000 and 2020. Drawing on the displacement model proposed by York (2012), a displacement coefficient of renewables at − 1 indicates a one-to-one basis displacement, whereby each unit of RE generated corresponds to one fewer unit of energy generated by fossil fuels. A displacement coefficient ranging between − 1 and 0 represents partial displacement. Conversely, a displacement coefficient of 0 denotes that the increase in RE exerts no displacement effect on fossil fuels.

The generation of electricity is one of the most crucial aspects of decarbonizing the energy sector. The IEA (2017) estimates that decarbonization of the energy sector, specifically through electricity generation, alone could reduce energy-related emissions by more than two-thirds under a “two-degree scenario,” largely through the widespread adoption of renewable technology. However, if the amount of electricity generated is only considered, one cannot gain a comprehensive understanding of the costs associated with capacity. By considering capacity, it is possible to accurately assess the investment decision by understanding the cost incurred to generate a specific quantity of electricity. Therefore, this study contributes to the existing body of literature by examining the correlation between renewable and fossil fuel generation technologies, with a focus on generation capacity rather than solely relying on generation data.

In this regard, the dependent variable is the fossil fuel generation capacity (Generationcap^FF^), and it encompasses the combined capacity of coal, oil, and natural gas power plants. The main explanatory variable is the renewable electricity generation capacity Generationcap^RE^). As in the related paper by Verdolini et al. (2016), the fossil fuel capacity and RE energy capacity are obtained by dividing the net generation capacity by the total generation capacity in the country *i* and time *t*. Total capacity is used as a rescaling variable to determine the share of energy sources. The data for fossil fuels and renewables are obtained from the U.S. Energy Information Administration (EIA). The other explanatory variable is technological innovation (*TI*). This variable is measured using the data on patent counts in clean energy technologies, which are (i) electrical machinery, apparatus and energy; (ii) environmental technology; and (iii) engines, pumps, and turbines. Patents possess the benefit of serving as a reliable indicator of innovative endeavors and exhibit a strong correlation with numerous alternative metrics of innovation. Technological innovation is a crucial element that directly contributes to the expansion of RE capacities. This is expected to accelerate the displacement of fossil fuels by renewables. The data for technological innovation comes from the WIPO statistics database. Additionally, gross domestic product (GDP) is included to capture overall economic performance. This data is obtained from the World Bank Database Indicator (WDI). Consequently, the general structure of the main empirical model, which is discussed above, is as follows:

1 $${{\text{Generationcap}}}_{it}^{{\text{FF}}}={\alpha }_{0}+{\alpha }_{1}{{\text{Generationcap}}}_{it}^{{\text{RE}}}+{\alpha }_{2}{LTI}_{{\text{it}}}+{\alpha }_{3}{LGDP}_{it}+{\varepsilon }_{it}$$

where ${{\text{Generationcap}}}_{it}^{{\text{FF}}}$ and ${{\text{Generationcap}}}_{it}^{{\text{RE}}}$ stand for fossil fuel generation capacity and renewable electricity generation capacity, respectively. ${TI}_{it}$ presents technological innovation and ${GDP}_{it}$ is the economic growth. ${\alpha }_{1}$ is the displacement coefficient, and ${\varepsilon }_{it}$ is the error term. *L* indicates that variables are logarithmically converted.

In the main empirical model, the various definitions of RE sources are employed separately for robustness checks. However, specifically variable RE sources, such as solar and wind power, are considered in Eq. (2). Even though their output varies depending on meteorological conditions, they are the most promising technologies among RE sources. More importantly, due to the rapid decline in their costs, they have become the primary option for new capacity additions on a global scale, challenging prevalent power systems like fossil fuel technologies. In this regard, the empirical model is rewritten as follows:

2 $${{\text{Generationcap}}}_{it}^{{\text{FF}}}={\alpha }_{0}+{\alpha }_{1}{{\text{Generationcap}}}_{it}^{{\text{VRE}}}+{\alpha }_{2}{LTI}_{it}+{\alpha }_{3}{LGDP}_{it}+{\varepsilon }_{it}$$

where ${{\text{Generationcap}}}_{it}^{{\text{VRE}}}$ denotes the installed variable RE capacity. Other variables remain as defined earlier. The assessment of Eq. (2) has been carried out utilizing data from 23 OECD^1^ countries, considering the availability of data for variable RE sources across the research period of the study. Natural logarithms are used for all variables.

Table 1 displays descriptive statistics for the whole sample in panel A and for the inclusive sample in panel B. According to panel A in Table 1, in terms of average values, the generation capacity of fossil fuels surpasses that of renewable electricity generation capacity. Specifically, the generation capacity of hydropower and biomass in total accounts for approximately two-thirds of the total generation capacity of renewables on average. The renewable electricity generation capacity ranges from 0 up to 95%, and the maximum value of fossil fuel generation is about the same as renewables. The generation capacity of RE sources exhibits a greater degree of leftward skewness in comparison to that of fossil fuels. Panel B of Table 1 shows that the mean value of fossil fuel generation capacity is 0.476, while the electricity generation capacity of variable renewables exhibits a lower mean, with a value of 0.103

**Table 1:** Descriptive statistics

Variables	Obs	Mean	Std. dev	Min	Max
Panel A. Whole sample (*N* = 36)					
Generationcap^FF^	756	0.517	0.248	0.004	0.999
Generationcap^RE^	756	0.318	0.225	0.001	0.959
Generationcap^RE^ (excl. hydro)	756	0.115	0.114	0	0.548
Generationcap^RE^ (excl. hydro, waste, and biomass)	756	0.089	0.101	0	0.469
Generationcap^HYDRO^	756	0.203	0.226	0.003	0.950
Generationcap^BIOMASS^	756	0.027	0.031	0	0.142
*LTI*	756	6.134	2.221	0.010	11.181
*LGDP*	756	10.202	0.719	8.284	11.63
Panel B. Inclusive sample (*N* = 23)					
Generationcap^FF^	483	0.476	0.247	0.004	0.928
Generationcap^VRE^	483	0.103	0.111	0.001	0.467
Generationcap^SOLAR^	483	0.033	0.053	0	0.235
Generationcap^WIND^	483	0.071	0.08	0	0.360
*LTI*	483	6.790	2.026	2.197	11.181
*LGDP*	483	10.376	0.655	8.712	11.63

For macro-level variables, cross-sectional dependence (*CD*) can arise because of various factors, such as macroeconomic connections that manifest through (i) unobservable time-varying shocks, (ii) participation in institutional memberships, and (iii) the economic structure. As a result, *CD* has the potential to impact the accuracy and reliability of the analysis findings and neglecting this dependence could lead to biased and inconsistent estimations. Table 2 presents a summary of the findings from the Pesaran (2015) *CD* test and indicates that both Eqs. (1) and (2) exhibit the presence of a *CD* issue in their respective variables. In addition, the slope homogeneity (SH) test developed by Pesaran and Yamagata (2008) is used in this study. The results, as displayed in Table 3, all agree that there is slope heterogeneity present. Accordingly, to account for *CD* and heterogeneity, second-generation panel data methodologies are employed in the study.

**Table 2:** *CD* and unit root test results

Tests	Pesaran *CD*	CADF	
Variables	*CD* test	*I*(0)	*I*(1)
Panel A. Whole sample			
Generationcap^FF^	112.229***	− 1.376	− 3.812***
Generationcap^RE^	106.864***	− 1.755	− 3.392***
Generationcap^RE^ (excl. hydro)	108.997***	− 1.145	− 2.504***
Generationcap^RE^ (excl. hydro, waste, and biomass)	106.087***	− 1.224	− 2.367***
Generationcap^HYDRO^	113.250***	− 1.604	− 4.266***
Generationcap^BIOMASS^	103.556***	− 1.708	− 3.698***
*LTI*	104.152***	− 1.845	− 4.776***
*LGDP*	111.547***	− 1.679	− 2.684***
Panel B. Inclusive sample			
Generationcap^FF^	70.538***	− 1.876	− 3.572***
Generationcap^VRE^	67.834***	− 1.182	− 2.065**
Generationcap^SOLAR^	65.954***	− 0.579	− 2.809***
Generationcap^WIND^	68.792***	− 1.774	− 3.005***
*LTI*	67.277***	− 1.787	− 4.484***
*LGDP*	70.744***	− 1.497	− 2.134**

*** shows 1% significance level

**Table 3:** SH test results

	Whole sample		Inclusive sample	
	Delta	*p*-value	Delta	*p*-value
Δ	10.442	0.000	15.369	0.000
ΔAdj	13.814	0.000	18.823	0.000

The subsequent stage involves the determination of the stationary properties of the variables through the utilization of the CADF panel unit root test developed by Pesaran (2007). The findings from Table 2 indicate that all variables are non-stationary at the level, but they exhibit stationarity after being subjected to first differencing.

To account for the existence of *CD* and heterogeneously sloping properties within the panel dataset, the present study utilizes the CCE-MG approach developed by Pesaran (2006). One notable advantage of this estimator, in comparison to other panel estimators, is its ability to effectively account for variables that exhibit cross-sectional dependency, heterogeneous characteristics, and the existence of unit root (Chudik et al. 2011). The CCE-MG approach addresses these concerns by improving the country-specific regression equation in an effective way (Eberhardt 2012). The means of the dependent and independent variables at the cross-sectional level are incorporated into this equation. The CCE-MG model is represented by the following equation:

3 $${lny}_{it}={\alpha }_{it}+{\beta }_{i}{lnx}_{it}+{\gamma }_{i}ln{\overline{y} }_{it}+{\delta }_{i}ln{\overline{x} }_{it}+{c}_{i}{f}_{t}+{\varepsilon }_{it}$$

**Fig. 3:**
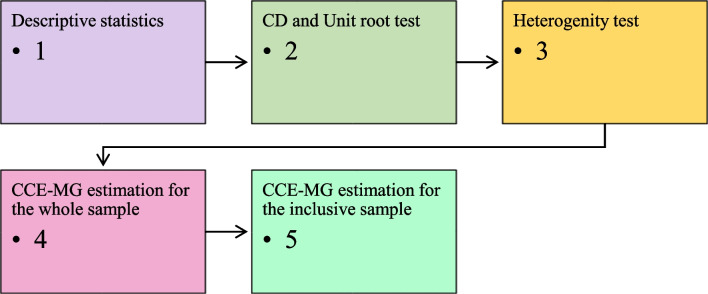
Empirical flowchart

In Eq. (4), the variables ${y}_{it}$ and ${x}_{it}$ are used to represent the subject variables. The parameter coefficients ${\beta }_{i}$ are estimated values, while ${f}_{t}$ represents an unobserved common factor that possesses heterogeneous characteristics and can be accounted for by a combination of ${\overline{y} }_{it}$ and ${\overline{x} }_{it}$. The variables ${\alpha }_{it}$ and ${\varepsilon }_{it}$ represent the group fixed effect and the error term, which encompass the unobservable factors, respectively. The sequence of the empirical approach used in the study is shown in Fig. 3. After testing the statistical, stationarity, *CD*, and homogeneity properties of the panel dataset, the study uses the CCE-MG estimator for whole and inclusive samples, respectively, in the fourth and fifth steps.

## Empirical results and discussion

Table 4 summarizes the main findings of the CCE-MG estimator using the above-mentioned Eq. (1) variations. Model 1 provides a specification that includes the electricity generation capacity of all forms of renewables.

**Table 4:** CCEMG results for whole sample

Variables	Dependent variable: Generationcap^FF^		
	Model 1	Model 2	Model 3
Generationcap^RE^	− 0.869***	-	-
Generationcap^RE^ (excl. hydro)	-	− 0.932***	-
Generationcap^RE^ (excl. hydro, waste, and biomass)	-	-	− 0.945***
Generationcap^HYDRO^	-	− 1.034***	− 1.050***
Generationcap^BIOMASS^	-	-	− 0.974***
*LTI*	− 0.005**	− 0.001**	− 0.002**
*LGDP*	− 0.001*	− 0.008*	− 0.013*
Constant	0.799***	0.851***	0.817***

***, **, and * denote 1%, 5%, and 10% levels of significance, respectively

The findings from Model 1 reveal that there is a statistically significant and good displacement of fossil-fuels by renewables. More specifically, a 1% increase in renewable electricity generation capacity is associated with a 0.87% decrease in fossil fuel capacity.

Model 2 uses the generation capacity of renewables, excluding hydropower. Given that hydropower is a mature technology for which the majority of the natural endowment has already been utilized, it is not included in the calculation of renewable electricity. Moreover, as stated by Popp et al. (2011), hydropower is a fast-responding dispatchable technology that is frequently used to meet peak demand. It is therefore distinct from the other forms of RE technologies in terms of its limitations and characteristics. When excluding hydropower, the findings indicate that there is a statistically significant and higher displacement of fossil fuels than obtained in Model 1. In other words, a 1% increase in the generation capacity of non-hydro renewables is related to a 0.93% decrease in the generation capacity of fossil fuel sourced electricity. On the other hand, the displacement coefficient for hydropower is − 1.034, which indicates that a one-to-one displacement of fossil fuels by hydropower has occurred. In other words, hydropower strongly displaces fossil fuels. This might be because hydropower is the largest energy source among renewables by generation and capacity (IEA 2022b). In addition, hydropower plants exhibit superior capability for swiftly adjusting their electricity generation levels in comparison to other alternative power plants like natural gas, coal, and nuclear. The high level of flexibility possessed by them allows them to promptly adapt to changes in demand and effectively counterbalance variations in supply originating from alternative electricity sources. This makes hydropower a significant substitute for displacing fossil fuel-fired sources, thereby highlighting its importance.

Model 3 considers the generation of non-hydro and biomass renewables. Biomass can be stored and burned when it is needed, making it a dispatchable fuel source. It is possible to co-fire biomass by combining it with natural gas and coal, and biomass can be fed into a variety of burners that are also capable of burning fossil fuels (Verdolini et al. 2018). Hence, including biomass in the calculation of renewable electricity generation may result in biased estimates. Accordingly, the findings of Model 3 reveal that the displacement coefficient (− 0.945) is statistically significant and different from − 1. It is indicated that a 1% increase in the electricity generation capacity of non-hydro-biomass renewables leads to a decrease of 0.94% in the generation capacity of fossil fuels. This implies that non-hydro-biomass renewables substantially displace fossil fuels. In addition, biomass has an almost one-to-one displacement effect on fossil fuels, with a coefficient of − 0.974. Biomass possesses considerable potential as it can be utilized for direct combustion to generate heat or electricity, and it can also be transformed into alternative forms of oil or gas. Because of this potential, biomass can effectively compete with fossil fuels and displace them.

Overall, the findings obtained from Models 1 to 3 show that the displacement coefficients are very close to − 1 and the displacement coefficients for the different definitions of renewables significantly differ from each other in Models 1 to 3. The results indicate that RE sources are progressively becoming the default option due to their cost-effectiveness, and they are moving in the direction of displacing fossil fuel sources on a one-to-one basis. Over the past decade, there has been a significant decline in the cost of RE, and this can be attributed to gradual advancements in technologies, the realization of economies of scale, and the establishment of competitive supply chains. In 2020, approximately 162 GW of new generation capacity from renewables had costs lower than the least expensive form of new fossil fuel-based capacity. This accounts for around 62% of the net increase in renewable power generation globally (IRENA 2021). Accordingly, the growing competitiveness, and declining costs, coupled with the ongoing fossil fuel crisis, suggest that there is potential for renewables to completely replace fossil fuels soon, primarily driven by the expansion of their generation capacities. It can also be said that renewables possess the capacity additions to compensate for electricity generation derived from fossil fuels, thereby enabling the OECD countries, dependent on fossil fuels, to significantly diminish their reliance on such resources. This, consequently, helps to ensure a stable energy supply and meet the goals of cutting emissions. Moreover, the capacity additions of renewables for electricity generation have served to decarbonize the energy sector and limit the environmental damage caused by fossil fuels. It is also observed that hydropower and biomass exhibit more efficacies in competing with fossil fuels and displace a larger proportion of fossil fuels. This might be because dispatchable and mature RE technologies, such as hydropower and biomass, have the potential to provide electricity at a lower cost in comparison to fossil fuels, especially in regions where untapped economic resources remain available.

Table 5 summarizes the findings of the CCE-MG estimates using the above-mentioned Eq. (2) variations. In this context, Model 1 encompasses a specification that includes generation capacity for variable renewables, while Models 2 and 3 contain solar and wind power generation capacity. The findings from Table 5 show that the capacity of variable renewables is negatively associated with fossil fuel capacity, indicating the partial displacement of fossil fuels by variable renewables. Moreover, solar capacity has a negative effect on fossil fuel capacity, with a displacement coefficient of − 0.49. Regarding wind capacity, the displacement coefficient is negative and statistically significant (− 0.55), and it is slightly higher than solar capacity. The findings show that wind power seems to displace more fossil fuels than solar power does. In addition, the generation capacity of variable renewables has a relatively smaller displacement effect on fossil fuels than other types of renewables. Wind and solar power are prominent renewable energy sources that are expected to exert a growing influence on the future energy composition as a result of their declining costs. However, the cost reductions of these sources alone are insufficient to achieve the swift decarbonization of the power industry. This may be attributed to the fact that the yields of variable renewable technologies exhibit daily and seasonal fluctuations, which may also be associated with variations in energy demand and the cost of buying electricity from the grid. Overall, these results indicate that there is a significant pathway that still needs to be taken for OECD countries to attain one-to-one displacement of fossil fuels with variable renewables. Hence, OECD countries should prioritize these sources in their energy agendas to maximize the advantages of the sustainable structure of solar and wind power technologies.

**Table 5:** CCEMG results for inclusive sample

	Dependent variable: Generationcap^FF^		
	Model 1	Model 2	Model 3
Generationcap^VRE^	− 0.524***	-	-
Generationcap^SOLAR^	-	− 0.489***	-
Generationcap^WIND^	-	-	− 0.556***
*LTI*	− 0.003**	− 0.002**	− 0.011**
*LGDP*	− 0.043*	− 0.070*	− 0.012*
Constant	0.284*	0.497**	0.125*

Results based on panel data for 23 nations for which data is available

***, **, and * denotes 1%, 5% and 10% levels of significance, respectively

## Conclusion and policy implications

The burning of fossil fuels is the principal contributor to climate change; therefore, adjusting the energy composition is essential to attaining carbon neutrality and alleviating the adverse impacts of climate change. To address climate change, it is crucial to decrease the consumption of these carbon-intensive fuels, and significant sectors of the economy must undergo a transition away from reliance on fossil fuels. Recent global events have highlighted the significance of connecting the expansion of RE capacity with endeavors to accelerate the energy transition. This connection is crucial for enhancing the resilience, inclusivity, and climate resilience of the energy system. In fact, RE sources that are cost-competitive have a significant role in addressing the current energy and climate crises. They can expedite the transition towards meeting the goals outlined in the Paris Agreement, specifically the target of limiting global warming to 1.5 °C.

In this context, this study investigates the displacement effect of disaggregated RE sources on fossil fuels by employing panel data for 36 OECD countries spanning from 2000 to 2020, with the objective of evaluating the current state of the global energy transition. In this regard, the CCE-MG method is implemented. The results show that RE has a significant displacement impact on fossil fuels. Specifically, to effectively displace 1% of fossil fuel generation capacity, it is necessary to achieve an average increase of 1.15% in renewable electricity generation capacity in OECD countries. This observation remains consistent across a range of rigorous tests, which include varying interpretations of renewable generation capacity and the application of the CCE-MG estimation method. Excluding hydropower, biomass, and waste from the RE mix makes it much clearer that RE has a substantially greater influence on fossil fuel displacement. Under this scenario, on average, a displacement of 1% of fossil fuel necessitates 1.07% of renewable electricity. More precisely, a one-to-one displacement of fossil fuels occurs with hydropower and biomass. Further examination focusing on variable RE, utilizing panel data from 23 OECD countries, reveals that an average increase of 1.9% of variable renewable electricity is required to displace 1% of fossil fuel generation capacity.

Previous studies have predominantly examined the effects of renewables or fossil fuels on emissions. However, this study takes a different approach by investigating to what extent renewables can replace fossil fuels. It also compares the displacement impact of various forms of renewables within a dynamic framework, which provides a more comprehensive analysis. In this regard, this study adds to the existing body of literature by illustrating that the energy transition in OECD countries is well underway. It is also evident that RE sources are progressively engaging in direct competition with fossil fuels, achieving the potential displacement of the latter. The research underscores the potential of RE sources, particularly hydropower and biomass, in supplanting fossil fuels. The findings indicate that the capacity of renewables to displace fossil fuels can be partially attributed to their implementation in nations that have adopted measures aiming to combat climate change. Indeed, the actualization of this energy transition to RE sources depends on the continued dedication of countries to support the utilization of RE and related technologies.

Given the current unavailability of cost-effective technologies, the deployment of RE sources has emerged as a crucial aspect of the contemporary energy system, as these resources present the most competitive alternatives to fossil fuels. Effectively transitioning to renewables not only necessitates the advancement of RE technologies but also requires coordinated economic and political efforts for the successful execution of innovative energy production techniques derived from renewables. While renewables have improved cost competitiveness, notably wind and solar, government policies should adapt to the evolving market dynamics. For example, market-based mechanisms, such as fuel taxes and incentives, can promote the adoption of alternative energy sources while concurrently mitigating the rebound effect linked with fossil fuel consumption. Carbon and fuel tax implementation can generate revenue that can support the subsidization of RE sources as a viable displacement for fossil fuels. Furthermore, the phased reduction of subsidies in the fossil fuel market has the potential to serve as an effective climate policy measure aimed at accelerating the process of transitioning to alternative energy sources. Moreover, cost savings resulting from efficiency improvements can make government interventions focused on reducing energy demand and encouraging RE sources more economically viable, thus enhancing the probability of their effective execution. Given the declining costs associated with renewables, there exists a significant potential for a substantial portion of the future power supply to be derived from these sources. Hydropower, biomass, solar, and wind energy sources should be mostly utilized as economically viable alternatives to fossil fuel-fired power generation in regions with favorable resource availability and cost structures. In addition, the extremely short project lead times of wind and solar power make them crucial pillars in countries’ efforts to rapidly decrease their reliance on fossil fuels and eventually eliminate them altogether. In summary, fostering the deployment of RE sources, coupled with the establishment of climate change mitigation strategies, can expedite the transition of OECD countries towards a low-carbon economy.

Despite this study focuses on fossil fuel displacement with various forms of renewable energy, it has a few limitations. First, the study concentrates on OECD countries, where fossil fuel sources dominate the energy balance. Future research can analyze the displacement impact of renewables on EU countries or individual countries. Second, this study considers the displacement of fossil fuels with renewables, while ignoring nuclear energy. Hence, future studies can assess the displacement of fossil fuels with nuclear energy based on the availability of data. Third, the present study employs second-generation panel data approaches in order to investigate fossil fuel displacement with renewables. Future research can be conducted with recently developed methodologies, such as half-panel jackknife estimator.

## Data Availability

Data will be made available on request.

## Footnotes

1 The inclusive sample includes Australia, Austria, Belgium, Canada, Denmark, Finland, France, Germany, Greece, Italy, Japan, Luxembourg, Mexico, Netherlands, Norway, Portugal, Korea, Spain, Sweden, Switzerland, Turkey, the UK, and the U.S.
